# Nitrogen fixation and *nifH* diversity in human gut microbiota

**DOI:** 10.1038/srep31942

**Published:** 2016-08-24

**Authors:** Katsura Igai, Manabu Itakura, Suguru Nishijima, Hirohito Tsurumaru, Wataru Suda, Takumi Tsutaya, Eriko Tomitsuka, Kiyoshi Tadokoro, Jun Baba, Shingo Odani, Kazumi Natsuhara, Ayako Morita, Minoru Yoneda, Andrew R. Greenhill, Paul F. Horwood, Jun-ichi Inoue, Moriya Ohkuma, Yuichi Hongoh, Taro Yamamoto, Peter M. Siba, Masahira Hattori, Kiwamu Minamisawa, Masahiro Umezaki

**Affiliations:** 1 Graduate School of Biomedical Sciences, Nagasaki University, Nagasaki, Japan; 2 Department of Human Ecology, Graduate School of Medicine, The University of Tokyo, Tokyo, Japan; 3 Department of International Health, Institute of Tropical Medicine, Nagasaki University, Nagasaki, Japan; 4 Department of Environmental Life Sciences, Graduate School of Life Sciences, Tohoku University, Miyagi, Japan; 5 Department of Computational Biology and Medical Sciences, Graduate School of Frontier Sciences, The University of Tokyo, Chiba, Japan; 6 Center for Omics and Bioinformatics, Graduate School of Frontier Sciences, The University of Tokyo, Chiba, Japan; 7 Department of Integrated Biosciences, Graduate School of Frontier Sciences, The University of Tokyo, Chiba, Japan; 8 Department of Sociological Studies, Faculty of Human Sciences, Wako University, Tokyo, Japan; 9 Faculty of Letters, Chiba University, Chiba, Japan; 10 The Japanese Red Cross Akita College of Nursing, Akita, Japan; 11 Papua New Guinea Institute of Medical Research, Eastern Highlands Province, Papua New Guinea; 12 School of Applied and Biomedical Sciences, Federation University Australia, Victoria, Australia; 13 Japan Collection of Microorganisms, RIKEN BioResource Center, Ibaraki, Japan; 14 Department of Life Science and Technology, Tokyo Institute of Technology, Tokyo, Japan

## Abstract

It has been hypothesized that nitrogen fixation occurs in the human gut. However, whether the gut microbiota truly has this potential remains unclear. We investigated the nitrogen-fixing activity and diversity of the nitrogenase reductase (NifH) genes in the faecal microbiota of humans, focusing on Papua New Guinean and Japanese individuals with low to high habitual nitrogen intake. A ^15^N_2_ incorporation assay showed significant enrichment of ^15^N in all faecal samples, irrespective of the host nitrogen intake, which was also supported by an acetylene reduction assay. The fixed nitrogen corresponded to 0.01% of the standard nitrogen requirement for humans, although our data implied that the contribution in the gut *in vivo* might be higher than this value. The *nifH* genes recovered in cloning and metagenomic analyses were classified in two clusters: one comprising sequences almost identical to *Klebsiella* sequences and the other related to sequences of Clostridiales members. These results are consistent with an analysis of databases of faecal metagenomes from other human populations. Collectively, the human gut microbiota has a potential for nitrogen fixation, which may be attributable to *Klebsiella* and Clostridiales strains, although no evidence was found that the nitrogen-fixing activity substantially contributes to the host nitrogen balance.

Nitrogen fixation, the reduction of atmospheric dinitrogen to ammonium, is a process that changes a biologically inactive form of nitrogen to a compound that can be readily used by organisms^1^. Diverse microbial communities use this process to fulfil their nitrogen demands. For example, the microbiota associated with invertebrates living in nitrogen-poor environments occasionally include diazotrophs (nitrogen-fixing prokaryotes). Nitrogen fixation in these animals, including termites^2^, wood-boring beetles^3^, shipworms^4^, sponges^5^, and corals^6^, has been tested by the measurement of ^15^N_2_ incorporation and of reduction of acetylene, an analogue of dinitrogen, and recently also by nanoscale secondary ion mass spectrometry (NanoSIMS). The phylogenetic diversity of the nitrogenase reductase (NifH) gene, a molecular marker of nitrogen fixation, has also been extensively examined in the symbiotic microbiota of various invertebrates^2^,^3^,^5^,^7^.

In contrast, there have been few studies of nitrogen fixation in vertebrates. Several reports have been published, but almost exclusively in the 1970s. No or only slight acetylene-reducing activity was detected in the rumen contents or faeces of herbivorous mammals, such as sheep^8^,^9^,^10^, goats^8^,^10^, cows^11^,^12^, rabbits^8^, and reindeer^8^. *In situ* examinations of nitrogen fixation were conducted in a sheep^13^ and a guinea pig^14^. In the sheep, acetylene gas was injected into the rumen of a live animal, and acetylene-reduction rates of 0–5.1 nmol ethylene/ml rumen gas/h were detected. The live guinea pig was maintained in an atmosphere containing acetylene, and 75 nmol of ethylene was detected in the rearing chamber after 22 h.

The human distal gut is an anaerobic environment, like those of other mammals, where dinitrogen accounts for 60–80% of the gaseous content^15^. In a study of people with a low-nitrogen diet in Papua New Guinea (PNG), Bergersen and Hipsley (1970) hypothesized that nitrogen fixation occurs in the human distal gut^14^. The habitual nitrogen intake of the study subjects was estimated to be 49–74 mg/kg body weight/day in 1967^16^, which is much lower than the estimated average requirement for humans (105 mg/kg body weight/day)^17^. Nevertheless, these individuals displayed no disorders associated with nitrogen (protein) deficiency^18^. Bergersen and Hipsley collected faeces from an unspecified number of PNG individuals and five Europeans, and analysed them with an acetylene reduction assay. They detected 0.75 nmol/g/h acetylene-reducing activity in one of the European faecal samples and ‘slight but significant’ activities in all the PNG samples. However, the details were not described, and the samples were supplemented with glucose, which may have altered their acetylene-reducing activity. Because there have been no subsequent studies, whether the human gut microbiota truly has a capacity for nitrogen fixation, and if it does, whether it is specific to individuals with low nitrogen intake, remain unclear.

The diet of certain PNG individuals is still composed mostly of sweet potatoes today, resulting in a below-standard nitrogen intake^19^, even though their average nitrogen intake has been increasing since the 1970s. Therefore, we collected faecal samples from PNG individuals with low to sufficient nitrogen intake, and from Japanese individuals, with much higher nitrogen intake. To detect the nitrogen-fixing activity of the faecal microbiota and evaluate the effects of the habitual nitrogen intake, the ^15^N_2_ incorporation and acetylene reduction by the faecal samples were tested. We also examined the phylogenetic diversity of the *nifH* gene in the faecal samples by analysing clone libraries of *nifH* genes and transcripts and with metagenomic sequencing. In addition to *nifH*, the nitrogenase (NifDK) genes were identified in the PNG and Japanese metagenomes and in publicly available databases of human faecal metagenomes from other populations. This study demonstrates the nitrogen-fixing activity of the human faecal microbiota and presents a list of candidate nitrogen-fixing bacteria in the human gut.

## Results

### Nitrogen-fixing activity

Table 1 lists the faecal samples used in this study, with information on the PNG and Japanese host individuals, including their habitual nitrogen intake. Most samples were collected from males, because it has been suggested that nitrogen requirements fluctuate during the menstrual cycle and pregnancy in females^20^,^21^. To measure nitrogen-fixing activity, we selected six PNG (V001–035) and two Japanese (J001 and 002) individuals, so that the host nitrogen intake ranged from below to above (67.3–209.1 mg/kg body weight/day) the standard requirement (105 mg/kg body weight/day)^17^ (Table 1). Frozen faecal samples from these individuals were subjected to a ^15^N_2_ incorporation assay.

All the faecal samples from both the PNG and Japanese individuals incorporated ^15^N_2_ gas, whereas faecal samples sterilized by autoclaving did not (Table 2). The increases in the δ^15^N values (Δδ^15^N) ranged from 0.68‰ to 1.26‰, and were statistically significant (Student’s *t* test or Welch’s *t* test: *p* < 0.05, n = 3). These values corresponded to nitrogen fixation rates of 0.065–0.147 nmol/g wet faeces/h (Supplementary Table S1). There was no correlation between Δδ^15^N and the nitrogen intake of the host (Pearson’s product-moment correlation: *r* = 0.22; Spearman’s rank correlation: *ρ* = 0.21). The acetylene reduction assay supported the occurrence of nitrogen fixation in the two Japanese samples with high nitrogen intake (209.1 and 183.2 mg/kg body weight/day). The emission of ethylene was observed in the presence of acetylene (Supplementary Figure S1), and the nitrogen fixation rates were estimated to be 0.008 and 0.143 nmol/g/h, when the theoretical reduction ratio C_2_H_2_:N_2_ ≈ 3^22^ was used.

**Figure 1:**
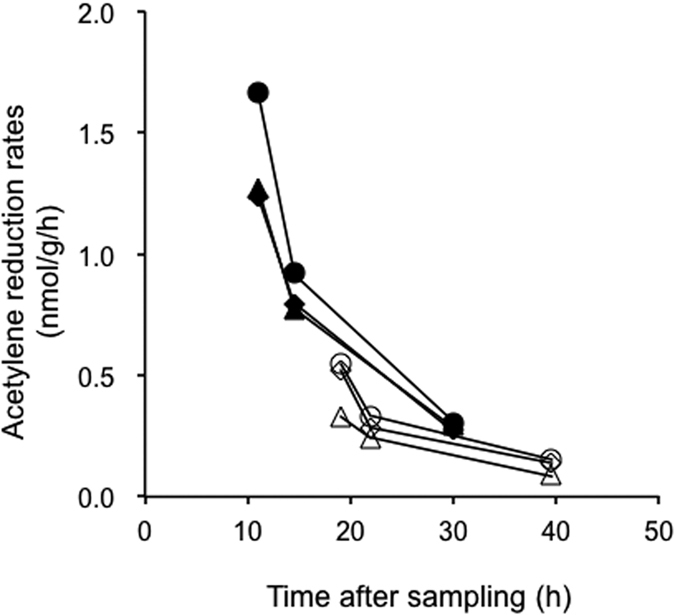
Decline in the acetylene-reduction rates in unfrozen Japanese faecal samples, N001-1 and N003. The experiments were performed in triplicate. Closed symbols, N001-1; open symbols, N003.

To further confirm the occurrence of nitrogen fixation in the faecal samples of human individuals with high nitrogen intake, we additionally examined the acetylene-reducing activities in six faecal samples from five Japanese individuals (N001–005) (Table 1). In this assay, each faecal sample was divided into two: one was analysed directly (‘unfrozen sample’) and the other was frozen before analysis (‘frozen sample’). By comparing these two types of sample, we also evaluated the effect of freezing on the nitrogen-fixing activity. All the unfrozen samples showed acetylene-reducing activities, ranging 0.055–1.608 nmol/g/h, which were 1.6–9.5 times higher than the values for the corresponding frozen samples (Table 3). The differences were statistically significant for samples N001-1 and N003 (Welch’s *t* test: *p* < 0.01, n = 3). We also observed that the acetylene-reducing activity declined within 24 h of sampling (defecation) (Fig. 1). Furthermore, the acetylene-reduction rates correlated negatively with the time after defecation in the unfrozen samples (Pearson’s product-moment correlation: *r* = −0.86), although the host difference might have affected the data (Table 3).

### PCR and reverse transcription (RT)–PCR amplification of *nifH*

**Figure 2:**
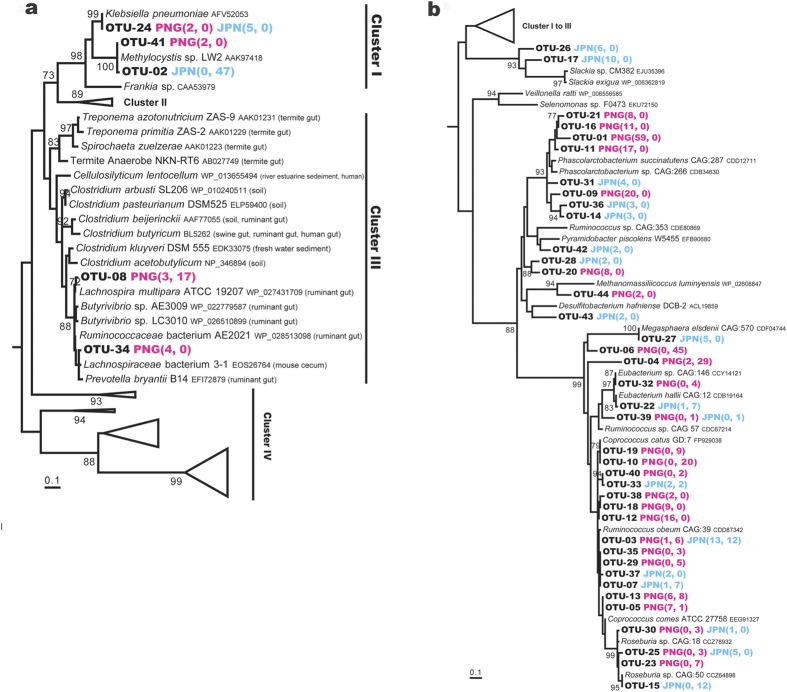
Phylogenetic positions of *nifH* recovered from Papua New Guinean and Japanese faecal samples, based on the deduced amino acid sequences. (**a**) Sequences affiliated with *nifH* Clusters I and III. (**b**) Sequences affiliated with *nifH* Cluster IV. Numbers in parentheses indicate the numbers of clones of genes and transcripts affiliated with the OTUs. A total of 113 amino acid positions were used, corresponding to positions 45–157 of the *Klebsiella pneumoniae* NifH sequence (AFV52053). 100 bootstrap resamplings were performed. Chlorophyllide reductase subunit BchX of *Rhodobacter sphaeroides* (CAB38747) was used as an outgroup. Only bootstrap confidence levels ≥70% are shown. OTU-02 and OTU-41 are most probably sequences contaminating in reagents.

DNA and RNA were extracted from each of the RNA*later*^®^-preserved faecal samples from six PNG individuals and four Japanese individuals (Table 1). PCR and RT–PCR were performed with universal primers for *nifH*^23^. Genes and transcripts of the predicted size, around 360 base pairs (bp), were detected in all the samples. A total of 259 genes and 265 transcripts encoding NifH homologues were detected and sorted to 44 operational taxonomic units (OTUs) (Supplementary Table S2). These were phylogenetically affiliated with *nifH* Cluster I (3 OTUs, 56 clones), Cluster III (2 OTUs, 24 clones), or Cluster IV (39 OTUs, 444 clones) (Fig. 2), according to the classification of *nifH* sequences by Zehr *et al.*^24^

Cluster I is composed of *nifH* sequences from diverse proteobacteria and cyanobacteria^24^. The OTU-24 sequence was recovered as gene clones from the PNG and Japanese samples (Fig. 2a). The nucleotide sequences were nearly identical (99%) to the *nifH* sequences of *Klebsiella* species, such as *Klebsiella pneumoniae*. The OTU-02 and OTU-41 sequences were almost identical to known contaminants frequently detected in PCR reagents^25^, and were not detected in the metagenomic datasets described below. Therefore, it is likely that these were not true constituents of the *nifH* community in the faecal samples.

Cluster III is known to contain *nifH* sequences of anaerobes from various environments. OTU-08 and OTU-34 showed high amino acid sequence similarities to NiH of anaerobic bacteria isolated from mammalian intestinal tracts (Fig. 2a). OTU-08 was recovered as both genes and transcripts from a PNG faecal sample. This OTU shared 98% amino acid sequence identity with NifH of *Lachnospira multipara* and also 97% identity with those of *Butyrivibrio* species and Ruminococcaceae bacterium AE2021. These bacteria have been isolated from ruminant foreguts, according to the description in the sequence database. OTU-34 was recovered as gene clones from two PNG samples. This OTU shared more than 97% amino acid sequence identity with NifH of Lachnospiraceae bacterium 3-1 isolated from a mouse cecum and Ruminococcaceae bacterium AE2021 and *Prevotella bryantii* isolated from ruminant foreguts.

Diverse and abundant sequences were assigned to Cluster IV, which is also called the ‘NifH-like cluster’. The function of Cluster IV NifH is largely unknown, but it has been suggested to play a role other than in nitrogen fixation^24^,^26^. Most of the 39 OTUs showed high amino acid sequence similarities to NifH-like sequences of members of the order Clostridiales, belonging to genera such as *Ruminococcus*, *Coprococcus*, and *Eubacterium* (Fig. 2b).

### Detection of *nifH* Cluster I and III sequences with quantitative PCR (qPCR)

Because the abundant Cluster IV *nifH* sequences probably interfered with the detection of Cluster I and III sequences in the clone analysis discussed above, we also performed a qPCR analysis of nine PNG and four Japanese faecal samples (Table 4) to specifically detect sequences of OTU-24, OTU-08, and OTU-34. The detection limit was 1.25 × 10^4^ copies per one gram of faecal sample. Of the 13 samples tested, 10 showed the presence of at least one of these OTUs.

OTU-24 of Cluster I was detected in samples V004 and J002, as in the clone analysis, and also in another two PNG samples, L005 and V001. The copy number of the OTU-24 sequence ranged from 10^5^ to 10^6^ per gram wet faeces in the three PNG samples, whereas the Japanese sample J002 contained 10^9^ copies per gram wet faeces (Table 4). OTU-08 of Cluster III was detected in five PNG samples, including L005, in which the OTU was found with the clone analysis. OTU-34 was detected more widely, in six PNG and three Japanese samples, including samples L005 and V014, in both of which the OTU was also found in the clone analysis. The copy numbers of the OTU-08 and OTU-34 sequences ranged from 10^6^ to 10^8^ per gram wet faeces in the PNG samples, while 10^5^ to 10^6^ in the Japanese samples (Table 4).

### Metagenomic analysis

The faecal DNA samples from 16 PNG and two Japanese individuals, including the six PNG and two Japanese individuals tested in the ^15^N_2_ incorporation assay, were subjected to whole-genome shotgun sequencing (Supplementary Table S3). To facilitate homology searches, non-redundant gene sets were prepared from the metagenomic contigs from the PNG and Japanese samples. A published metagenomic dataset^27^ was incorporated into the Japanese set.

**Figure 3:**
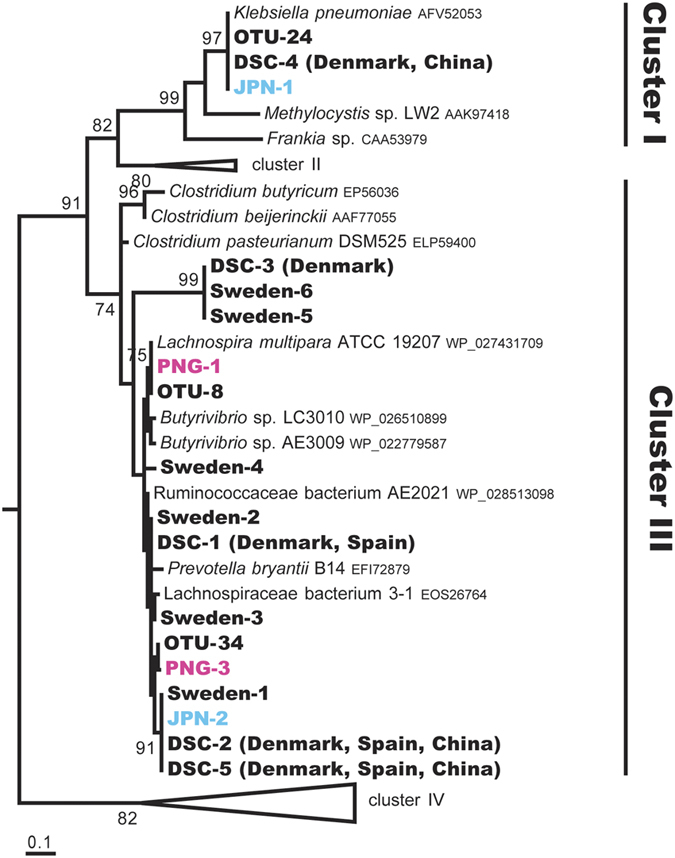
Phylogenetic positions of *nifH* from the human faecal metagenomes from six countries, based on deduced amino acid sequences. A total of 112 amino acid positions were used, corresponding to positions 45–157 of the *Klebsiella pneumoniae* NifH sequence (AFV52053). Of the four *nifH* genes from Papua New Guinean individuals, PNG-2 and -4 were not included because they corresponded to positions 138–283 and 124–264, respectively. ‘DSC’ indicates a combined non-redundant gene set of Danish, Spanish, and Chinese populations. See also the legend to Fig. 2.

In the PNG non-redundant gene set, four *nifH* homologues were identified and designated PNG-1–4 (Supplementary Table S4). These were affiliated with *nifH* Cluster III, sharing 94–98% amino acid sequence identities with NifH of *Lachnospira* and *Butyrivibrio* species. PNG-1 and PNG-3 showed 100% amino acid sequence similarity to OTU-08 and OTU-34, respectively (Fig. 3). The PNG-2 and PNG-4 sequence regions did not overlap most regions of the OTU sequences, and are therefore not shown in Fig. 3. In the Japanese gene set, two NifH homologues, designated JPN-1 and JPN-2, were identified and affiliated with *nifH* Clusters I and III, respectively (Fig. 3). JPN-1 shared 98% amino acid sequence identity with OTU-24, and JPN-2 shared 99% with OTU-34.

We also searched for *nifH* sequences in non-redundant gene sets constructed from publicly available databases of faecal metagenomes from other human populations, each consisting of 291 Danish^28^,^29^, 39 Spanish^28^, 145 Swedish^30^, or 363 Chinese^31^ individuals. In total, 11 NifH homologues were identified. Ten were assigned to *nifH* Cluster III, sharing 83–100% amino acid sequence identity with the amino acid sequences of Clostridiales species (*Lachnospira* and *Butyrivibrio* species) and also of OTU-08 and OTU-34 (Fig. 3 and Supplementary Table S4). The remaining homologue shared an identical amino acid sequence with NifH of *Klebsiella* species and OTU-24 in *nifH* Cluster I. Cluster III sequences were detected in 31.7%, 21.6%, 17.9%, and 10.7% of the Swedish, Danish, Spanish, and Chinese individuals, respectively, when 10^6^ sequencing reads were randomly chosen from each population. *Klebsiella*-related Cluster I sequences were detected in 0.7% and 6.6% of Danish and Chinese individuals, respectively.

The sequencing reads of the faecal metagenomes of the PNG and Japanese individuals who were tested with the ^15^N_2_ incorporation assay were further searched for *nifH*, using MG-RAST v3.2^32^. The results were consistent with the data presented above (Supplementary Table S5). We also searched for genes encoding the nitrogenase Mo-Fe protein subunits NifD and NifK in all the non-redundant gene sets as well as the sequencing reads of the PNG and Japanese, used in the search for NifH. NifD and NifK were encoded in all the datasets and shared high amino acid sequence identities with those of *Klebsiella* and members of the Clostridiales (Supplementary Tables S6 and S7).

## Discussion

We have demonstrated that the human gut microbiota truly has a capacity for nitrogen fixation, using ^15^N_2_ incorporation and acetylene reduction assays. The nitrogen-fixing activities were detected irrespective of the host habitual nitrogen intake; even the samples from Japanese individuals with nitrogen intake much higher than the standard requirement exhibited the activity. In addition, *nifHDK* genes were detected in the faecal metagenomes of PNG, Japanese, Chinese, and European individuals. Collectively, we suggest that a capacity for nitrogen fixation is maintained in various human populations.

The acetylene-reduction rates in the human faecal samples were up to 1.6 nmol/g/h, which is comparable to those detected in herbivorous mammals, such as sheep (0–1.4 nmol/g/h in the rumen)^8^,^9^,^10^, a rabbit (0.38 nmol/g/h in the cecum)^8^, and a reindeer (0–0.77 nmol/g/h in faeces)^8^. The nitrogen fixed in the human faecal samples corresponded to 0.36 mg/kg gut content/day, and the contribution to the host nitrogen balance was estimated to be 0.01%, at most. Thus, the nutritional impact of nitrogen fixation should be low in humans. However, caution is needed. Our data suggest that the nitrogen fixation by the human faecal microbiota is sensitive to the time after sampling (Fig. 1 and Table 3), as seen in termites. The nitrogen-fixing activity in termites declines within several hours of sampling^33^,^34^,^35^,^36^. Therefore, the nitrogen-fixing activity in the human gut *in vivo* might be higher than the values estimated in this study. In addition, freezing considerably reduced the nitrogen-fixing activity; this activity of the PNG faecal samples was possibly underestimated, because the sampling required a long-distance transportation and freezing.

It is generally believed that nitrogen fixation is suppressed when available nitrogen sources, such as ammonia and nitrate, are sufficient in the environment. Therefore, it has been assumed that the concentration of nitrogen compounds in the human gut is too high to allow biological nitrogen fixation^26^. However, previous studies have reported plasticity in the regulation of nitrogen-fixing activity in natural environments. In aquatic environments, such as marine sediments and salt marshes, nitrogen fixation (acetylene reduction) has been detected under high concentrations of nitrogen compounds (e.g., millimolar levels of ammonia)^37^,^38^. A high demand for nitrogen and balance between nitrogen fixation and nitrification have been suggested to explain these examples. In another case, certain plant-associated diazotrophs are less sensitive to ammonia in the symbiotic phase than in the free-living phase, and contribute to the nutrition of the host^1^. Our data suggest that the human gut microbiota also contains members capable of fixing nitrogen, even when the nitrogen input is sufficient.

In both of the PNG and Japanese faecal samples, the *nifH* genes identified (except the Cluster IV *nifH*-like genes) were affiliated with two clades: those in one set were almost identical to the *nifH* genes of *Klebsiella* species, and these genes in the other set were closely related to those of Clostridiales members. This result is consistent between the cloning results and metagenomic analyses, and the *nifH* genes found in the European and Chinese faecal metagenomes also fell within these two phylogenetic groups. Thus, *Klebsiella* and Clostridiales species are the candidate nitrogen-fixers in the human gut, although the possibility that other bacterial lineages possess the *nif-*genes via horizontal gene transfer cannot be excluded. The total copy numbers of these *Klebsiella-* and Clostridiales-related *nifH* genes were 10^5^–10^9^ copies per gram wet faeces (Table 4) and not correlated with the host nitrogen intake (Table 1) or with the nitrogen-fixing activity (Table 2). These *nifH* copy numbers are comparable to those in other diazotrophic environments, such as corals (10^7^ copies per gram tissue)^7^ and the rhizosphere (10^5^–10^7^ copies per gram soil)^39^,^40^.

Many *Klebsiella* strains have been identified as active diazotrophs in various environments, including soil, water, and plants^41^. *Klebsiella* species are generally not predominant, but are widely distributed in the gut microbiota of humans^28^,^42^,^43^. Bergersen and Hipsley (1970) predicted that *Klebsiella* species are one of the nitrogen fixers present in the human gut microbiota^14^. They isolated three bacterial strains, provisionally identified as *Klebsiella aerogenes*, on a nitrogen-free medium, from human faecal samples and determined their ^15^N_2_-incorporating activities.

The nitrogen-fixing activities of various Clostridiales strains containing Cluster III NifH were demonstrated with an acetylene reduction or ^15^N_2_ incorporation assay^44^. However, the bacterial strains, such as *Lachnospira* and *Butyrivibrio* species, of which NifH sequences constitute a clade with those from the human faecal microbiota, were not tested for nitrogen-fixing activity. A computational study suggested that certain bacteria expressing Cluster III NifH are likely to reduce acetylene and azide, but not dinitrogen, because they lack a histidine residue in the NifD protein (His422 in *Azotobacter vinelandii*)^45^. However, ^15^N_2_ fixation has recently been reported in *Endomicrobium proavitum*, which lacks His422 but retains all cysteine residues in NifD^46^. Among the relevant members of the Clostridiales and Bacteroidales (Figs 2a and 3), *L. multipara* ATCC 19207, Ruminococcaceae bacterium AE2021, and *P. bryantii* B14 lack His422 in NifD, but all the essential cysteine residues are conserved, as in *E. proavitum.* The other members of Clostridiales in this clade retain both the histidine and cysteine residues. In the NifD homologues recovered from the human faecal metagenomes, both of those with and without the histidine residue were identified. None of the NifD homologues lacked the cysteine residues. Therefore, there is currently no reason to consider that these bacteria cannot fix dinitrogen.

This study has shown that the human faecal microbiota has a potential capacity for nitrogen fixation, and that this capacity may exist in a wide range of human populations. However, evidence that this capacity substantially contributes to the host nitrogen balance was not obtained; the ecological meaning of nitrogen fixation in the human gut remains to be clarified.

## Materials and Methods

### Ethics statement

The study, involving human participants, was approved by the Institutional Review Board at the Papua New Guinea Institute of Medical Research (1025), the Papua New Guinea Medical Research Advisory Committee (11.25), and the Research Ethics Committee at the Graduate School of Medicine, The University of Tokyo (3391). All the participants provided their written informed consent. All experiments were performed in accordance with the approved study protocols.

### Study population

PNG participants were recruited in the Levani area (Levani Valley, Hela Province, Papua New Guinea) in March 2012 and March 2013, and in the Maprik area (East Maprik, East Sepik Province) in August 2012. Dietary data were obtained with a semi-quantitative food frequency questionnaire developed in our previous study^19^, including demographic and anthropometric data. Nineteen healthy PNG individuals, aged between 15 and 40 years, with body mass indices (BMIs) ranging from 19.2 to 27.2 kg/m^2^, were selected for the study (Table 1 and Supplementary Table S3). Their nitrogen intake was calculated based on their protein intake using a nitrogen-to-protein conversion factor of 6.25 (nitrogen weight = protein weight/6.25)^47^.

The Japanese participants were recruited in Tokyo, Japan, between June 2013 and April 2016. Their dietary information was obtained with a brief self-administered diet history questionnaire^48^, and their age, height, and weight were self-reported. Nine individuals, with ages and BMIs similar to those of the PNG participants, were selected (Table 1).

### Sample collection in PNG

In Levani in 2012, faecal samples were collected by each participant in a plastic container, and a portion of approximately 500 mg of the faeces was immediately suspended in 2 ml of RNA*later*^®^ (Ambion) (Table 1). In Maprik in 2012 and in Levani in 2013, faecal samples were collected and immediately enclosed in an AnaeroPack™ (Mitsubishi Gas Chemical), placed in a cooler container, and brought to our research base in town (Table 1 and Supplementary Table S3), which took 7–8 h to drive and/or walk from the rural communities in Maprik and Levani. At the base, a portion of the faeces was suspended in RNA*later*^®^ as described above, and another portion of approximately 2.0 g was frozen in a liquid nitrogen dry vapor shipper. These samples were transported to our laboratory at The University of Tokyo, Japan, and stored at −80 °C until analysis.

### Sample collection in Japan

Faecal samples were collected in 2012 and 2013 in Tokyo (Table 1), and suspended in RNA*later*^®^ immediately after defecation by each participant. Frozen samples from two individuals were also prepared. The samples were enclosed anaerobically in an AnaeroPack™ by each participant, brought to the laboratory at the ambient temperature (around 15 °C), and stored at −80 °C until analysis. In 2016, additional faecal samples were collected from five individuals without freezing or treatment with RNA*later*^®^ (Table 1). The samples were enclosed in an AnaeroPack™ by each participant and were stored at 4 °C until analysis.

### ^15^N_2_ incorporation assay

The frozen faeces were divided (cracked) into pieces without thawing. The faecal pieces were placed in a vacuum desiccator (7 l), as duplicate or triplicate samples, at the ambient temperature. The gas phase was immediately replaced with argon (Ar) and subsequently adjusted to (60% [v/v] ^15^N_2_ [99.7% atoms] and 40% [v/v] Ar) (SI Science, Saitama, Japan). The samples were incubated at 37 °C for 48 h. Control experiments without ^15^N_2_ gas were also performed in duplicate or triplicate. Sterile controls were prepared by autoclaving the samples at 120 °C for 20 min and incubating them with ^15^N_2_ gas under the same conditions. The ^15^N abundance (δ^15^N) was measured with the DELTA V Advantage ConFlo IV system (Thermo Fisher Scientific) at SI Science. The total nitrogen mass concentration (%N) was measured with a Flash 2000 elemental analyser (Thermo Fisher Scientific) in the Isotope Ecology Laboratory at The University of Tokyo. The analytical standard deviation (SD) was approximately <0.2‰ for δ^15^N and <0.77% for %N. For samples analysed in triplicate, Student’s *t* test or Welch’s *t* test was performed based on the results of an *F*-test. The correlation between Δδ^15^N and the host nitrogen intake was determined with Pearson’s product–moment correlation coefficient (*r*) and Spearman’s rank correlation coefficient (*ρ*). The calculation of the ^15^N mass is shown in Supplementary Table S1.

### Acetylene reduction assay

Frozen faeces from samples J001 and J002 were cracked into approximately 1.0–2.0 g pieces and divided into glass vials (net capacity 8.7 ml). The headspace was replaced with nitrogen gas followed by the injection of acetylene gas at a final concentration of 15% (v/v). The vials were placed at 37 °C. Faecal samples incubated without acetylene gas and empty vials with only acetylene gas were also prepared as controls. An aliquot (0.2 ml) of the headspace gas was analysed to measure the ethylene concentrations from day 0 to day 3 at 40 °C using a GC7A gas chromatograph (Shimadzu) equipped with a Propack N column (80/100 mesh, 2 m in length, 2.2 mm in diameter; Shimadzu) and a flame ionization detector.

Unfrozen faeces from samples N001 to N005 were divided into approximately 1.0–2.0 g portions in glass vials in an anaerobic chamber. Half the vials were frozen at −80 °C for 10–12 h for comparisons with the corresponding unfrozen samples. Aliquots (0.125 ml) of the headspace gas from both the unfrozen and frozen samples were analysed at 3, 6, and 21 h after the addition of acetylene gas for samples N001-1 and N003, and at 1 and 3 h after the addition of acetylene gas for the other samples.

### DNA extraction

DNA was extracted from the faecal samples preserved in RNA*later*^®^ or from frozen samples, according to the previously described methods^49^,^50^, with modifications. The faecal samples in RNA*later*^®^ were diluted and washed twice with phosphate-buffered saline (PBS). Faecal aliquots of 4 mg were prepared and suspended in 300 μl of Tris-SDS (250 μl of 200 mM Tris-HCl, 80 mM EDTA, pH 9.0, and 50 μl of 10% SDS). To this suspension, 300 mg of glass beads (0.1 mm diameter) and 500 μl of Tris-EDTA (TE)-saturated phenol (Wako, Japan) were added, and the cells disrupted with a Shakemaster Auto (Biomedical Science, Tokyo, Japan) for 15 min. After centrifugation, the supernatant was subjected to phenol/chloroform/isoamyl alcohol extraction and isopropanol precipitation. The extracted DNA was suspended in 100 μl of TE.

A frozen faecal sample was treated with 15 mg/ml lysozyme at 37 °C for 1 h, with 60 units/ml purified achromopeptidase (Wako 015-09951, Japan) at 37 °C for 30 min, and then with 1% SDS and 1 mg/ml proteinase K at 55 °C for 1 h. The sample was subjected to phenol/chloroform/isoamyl alcohol extraction and isopropanol precipitation. The extracted DNA was treated with RNase A, and purified with polyethylene glycol precipitation to remove any residual protein. The purified DNA was suspended in 300 μl of TE.

### RNA extraction and cDNA synthesis

RNA was extracted from faecal samples preserved in RNA*later*^®^, using the RNeasy^®^ Mini Kit (Qiagen). The samples were washed in PBS, and 20 mg aliquots of faeces were combined with a mixture of 600 μl of the RLT buffer contained in the kit, 7 μl of β-mercaptoethanol, 100 μl of TE buffer, and 300 mg of glass beads, and disrupted with the Shakemaster Auto for 5 min. The subsequent procedure was performed according to the manufacturer’s guidelines. DNA was removed from the extracted RNA with the Turbo DNA-free Kit (Ambion) at 37 °C for 1 h. Reverse transcription was performed at 55 °C using the SuperScript III First Strand Synthesis System (Invitrogen) with 0.5 μM nifH3 primer^23^. Negative controls without reverse transcription were prepared for each sample.

### PCR and RT–PCR amplification, cloning, and sequencing

Genes and transcripts encoding NifH were amplified with PCR and RT–PCR, respectively, using primers specific for *nifH* under previously described conditions^51^, with modifications (Supplementary Methods). The PCR products were separated by agarose gel electrophoresis and those of approximately 360 bp in length were purified with the QIAquick Gel Extraction Kit (Qiagen), cloned into the pGEM^®^-T Easy Vector System (Promega), and used to transform *Escherichia coli* DH5α cells. Randomly chosen clones were sequenced with the T7 primer using the BigDye Terminator Cycle Sequencing Kit V3.1 (Applied Biosystems) on an ABI 3730 Genetic Analyzer (Applied Biosystems).

### Identification and phylogenetic analysis of *nifH*

The sequences obtained with PCR and RT–PCR were examined with BLASTX searches of the National Center for Biotechnology Information (NCBI) non-redundant database. The sequences that showed high similarities (E-value ≤ 10^–50^) to known *nifH* sequences and encoded the amino acid residues conserved in NifH^23^ were subjected to subsequent screening. The selected sequences were aligned with ClustalX2^52^, and a distance matrix was calculated with the DNADIST program in the PHYLIP package^53^. The nucleotide sequences were sorted to OTUs with a 96% similarity cut-off, using the mothur program^54^. A phylogenetic analysis was performed based on the deduced amino acid sequences, using a maximum likelihood method with the Le and Gascuel substitution model in MEGA 6.0^55^.

### Quantitative PCR

Specific PCR primers and TaqMan^®^ probes were designed for the *nifH* phylotypes OTU-08, OTU-24, and OTU-34 (Supplementary Table S8). Standard curves were constructed in triplicate using serial dilutions of linearized plasmids containing each target sequence. Quantitative PCR amplification was performed with the FastStart Essential DNA Probes Master (Roche Diagnostics) on the LightCycler Nano System (Roche) under cycling conditions: 95 °C for 10 min, and 40 cycles of 95 °C for 10 s and 60 °C for 30 s. Specific amplification was verified by cloning and sequencing the PCR products.

### Metagenomic analysis

Whole-genome shotgun sequencing was performed on an Ion PGM™ System with the Ion 318™ Chip Kit and Ion PGM™ 400 Sequencing Kit (Life Technologies). A total of 51,633,321 and 7,099,635 reads (average 3,227,083 and 3,549,818) were obtained for the 16 PNG and two Japanese samples (Supplementary Table S3), respectively, and a non-redundant gene set was constructed for each population. The method is described in detail in the Supplementary Methods.

Faecal metagenomic sequences for Danish^28^,^29^, Spanish^28^, Swedish^30^, and Chinese^31^ populations were retrieved from the NCBI archives. A combined non-redundant gene set for the Danish, Spanish, and Chinese populations was obtained at the GigaDB database (http://gigadb.org), and the non-redundant gene set for the Swedish population was provided by Fredrik Karlsson (Chalmers University of Technology, Sweden).

The amino acid sequences deduced from the non-redundant gene sets were searched for NifH homologues using BLASTP with a cut-off level of E-value ≤ 10^–5^, against *nifH* (K02588) genes from the Kyoto Encyclopedia of Genes and Genomes (KEGG) database. A supercomputer at the Human Genome Center, The University of Tokyo (http://sc.hgc.jp/shirokane.html) was used for the analysis. Sequences of more than 120 amino acids were incorporated into the *nifH* database in the ARB format created by Zehr’s laboratory (www.jzehrlab.com/#!nifh-database/c1coj) to classify the sequences into *nifH* Clusters^24^. The BLASTX search results for all the amino acid sequences containing ≤ 120 amino acids were also manually checked to eliminate Cluster IV NifH. The frequencies of the retrieved NifH homologues in each metagenomic dataset were calculated by mapping 10^6^ randomly chosen reads to the corresponding non-redundant gene sets, using the Bowtie2 tool^56^, with a criterion of 95% nucleotide sequence identity. The NifD and NifK sequences were identified with BLASTP using a cut-off of E-value ≤ 10^–5^ against NifD (K02586) and NifK (K02591) in the KEGG database, respectively. NifD and NifK sequences with more than 80% amino acid identity to those of bacteria containing Cluster I or Cluster III NifH were selected.

Additional searches for *nifH*, *nifD*, *and nifK* were made in the metagenomic sequence reads from the samples used for the ^15^N incorporation assay, using MG-RAST^31^. The method is described in detail in the Supplementary Methods.

## Additional Information

**Accession numbers**: The nifH sequences of the 41 OTUs were deposited at GenBank/EMBL/DDBJ under accession numbers, LC097013–56.

**How to cite this article**: Igai, K. *et al.* Nitrogen fixation and *nifH* diversity in human gut microbiota. *Sci. Rep.* **6**, 31942; doi: 10.1038/srep31942 (2016).

## Supplementary Material

Supplementary Information

## Figures and Tables

**Table 1:** PNG and Japanese sample information.

Sampling area and year	ID	Sex^a^	Age	BMI^b^	N intake^c^	Sample type^d^	Analyses^e^	
							Activity	*nifH*
PNG								
Levani (2012)	L005	M	23	22.9	85.2	R		✓
Levani (2012)	L006	M	35	22.8	89.6	R		✓
Levani (2012)	L015	M	20	21.2	69.6	R		✓
Levani (2013)	V001	M	39	22.9	103.4	Fro, R	✓	
Levani (2013)	V004	M	22	22.7	109.6	Fro, R	✓	✓
Levani (2013)	V009	M	35	24.3	117.2	Fro, R	✓	
Levani (2013)	V014	M	22	26.2	67.3	Fro, R	✓	✓
Levani (2013)	V022	F	35	27.2	87.3	Fro, R	✓	
Levani (2013)	V035	M	40	22.9	148.0	Fro, R	✓	✓
Japanese								
Tokyo (2013)	J001	M	44	25.1	209.1	Fro, R	✓	✓
Tokyo (2013)	J002	M	40	21.6	183.2	Fro, R	✓	✓
Tokyo (2014)	J004	M	31	22.9	273.3	R		✓
Tokyo (2014)	J005	M	24	22.2	210.3	R		✓
Tokyo (2016)	N001	M	25	24.4	170.9	Unfrozen	✓	
Tokyo (2016)	N002	M	23	22.7	176.4	Unfrozen	✓	
Tokyo (2016)	N003	F	41	19.2	250.0	Unfrozen	✓	
Tokyo (2016)	N004	M	39	24.6	177.1	Unfrozen	✓	
Tokyo (2016)	N005	F	38	19.7	197.6	Unfrozen	✓	

^a^M, male; F, female.

^b^BMI, body mass index, calculated as body weight (kg) per body height (m)^2^.

^c^Values are given as total N (mg) per body weight (kg) per day.

^d^R, preserved in RNA*later*; Fro, frozen.

^e^Activity, ^15^N_2_-incorporation and/or acetylene reduction assays; *nifH*, cloning analysis.

**Table 2:** Incorporation of ^15^N_2_ gas by PNG and Japanese faecal samples.

Sample ID	δ^15^N value^a^(‰)		Δδ^15^N^b^	*p* value^c^
	^15^N_2_ gas (+)	^15^N_2_ gas (−)		
PNG				
V001	5.90 ± 0.32	4.83 ± 0.10	**1.07**	0.0025
V004	4.26 ± 0.22	3.09 ± 0.38	**1.17**	0.0049
V009	6.17 ± 0.26	5.33 ± 0.04	**0.84**	0.0139
V014	6.44 ± 0.60	5.32 ± 0.05	**1.11**	0.0429
V022	6.32 ± 0.60	5.64 ± 0.05	**0.68**	0.0283
V035	3.74	2.66	**1.08**	N. A.
Japanese				
J001	7.48	6.22 ± 0.33	**1.26**	N. A.
J002	5.58 ± 0.18	4.78 ± 0.21	**0.80**	0.0041
Sterile control				
V004	3.22	3.09 ± 0.38	**0.13**	N. A.
V009	4.86	5.33 ± 0.04	**− 0.47**	N. A.
J002	4.66 ± 0.08	4.78 ± 0.21	**− 0.12**	0.2066

^a^Averages and SD of triplicate samples are shown. For duplicate samples, only average values are shown.

^b^Δδ^15^N = δ^15^N[^15^N_2_ (+)] − δ^15^N [^15^N_2_ (−)].

^c^Student’s *t* test for V001 and V004; Welch’s *t* test for the other samples. N. A., not applicable.

**Table 3:** Acetylene-reduction rates in Japanese faecal samples.

Sample ID	Time between sampling and assay^a^ (h)	Acetylene reduction rates (nmol/g/h)	
		Unfrozen	Frozen
N001-1^b^	8	1.390 ± 0.237	0.472 ± 0.109
N001-2^b^	6	1.608 ± 0.422	0.954 ± 0.010
N002	8	0.660 ± 0.403	N. A.
N003	15.5	0.466 ± 0.117	0.049 ± 0.034
N004	16.5	0.331 ± 0.127	0.082 ± 0.055
N005	27	0.055 ± 0.010	N. A.

^a^Time between defecation and the addition of acetylene to the unfrozen samples or freezing (the frozen samples).

^b^N001-1 and N001-2 were sampled from the same individual (N001) on different days.

**Table 4:** Detection of Cluster I and Cluster III *nifH* genes with qPCR.

ID	Target copy number (copies/wet faeces g)		
	OTU-24	OTU-08	OTU-34
PNG			
L005	8.4 × 10^4^	2.4 × 10^6^	3.1 × 10^7^
L006	UD	UD	1.4 × 10^6^
L015	UD	6.1 × 10^7^	1.4 × 10^8^
V001	1.1 × 10^5^	UD	UD
V004	5.1 × 10^6^	DNQ	UD
V009	UD	UD	UD
V014	UD	4.0 × 10^6^	3.9 × 10^7^
V022	UD	DNQ	1.4 × 10^6^
V035	UD	UD	9.6 × 10^7^
Japanese			
J001	UD	UD	DNQ
J002	1.6 × 10^9^	UD	1.4 × 10^6^
J004	UD	UD	UD
J005	UD	UD	1.8 × 10^5^

Underlining indicates OTUs detected in the cloning analysis of the corresponding samples. Each reaction was conducted in triplicate, and standards (10^1^–10^7^ plasmid copies) and negative controls were measured in duplicate. Quantification was performed when the target sequences were detected at ≥5 copies in at least two of the triplicate reactions. Abbreviations: UD, under detection limit; DNQ, detected but not quantified.
