# Supplementary material for: Nitrogen fixation and nifH diversity in human gut microbiota
Source: Sci Rep. 2016 Aug 24;6:31942. doi: 10.1038/srep31942 (PMC4995403; doi:10.1038/srep31942)
Supplement: Supplementary Information [file srep31942-s1.pdf]

Supplementary Information

Nitrogen fixation and *nifH* diversity in human gut microbiota

Katsura Igai, Manabu Itakura, Suguru Nishijima, Hirohito Tsurumaru, Wataru Suda, Takumi Tsutaya, Eriko Tomitsuka, Kiyoshi Tadokoro, Jun Baba, Shingo Odani, Kazumi Natsuhara, Ayako Morita, Minoru Yoneda, Andrew R. Greenhill, Paul F. Horwood, Jun-ichi Inoue, Moriya Ohkuma, Yuichi Hongoh, Taro Yamamoto, Peter M. Siba, Masahira Hattori, Kiwamu Minamisawa, Masahiro Umezaki

Supplementary Methods

Supplementary Table S1
Supplementary Table S2
Supplementary Table S3
Supplementary Table S4
Supplementary Table S5
Supplementary Table S6
Supplementary Table S7
Supplementary Table S8

Supplementary Figure S1

Supplementary Methods

PCR and RT-PCR conditions

The first-round amplification of nested PCR was conducted in 25 µl volume with 1 × *Ex Taq*® buffer, 0.2 mM dNTPs, 1.0 µM primers (nifH3 and nifH4)[1], 0.06 U *Ex Taq*® (Takara, Shiga, Japan), and 1 µl of DNA extract was added as a template for PCR and 2 µl of cDNA for RT-PCR. Amplification was performed under the conditions: 2 min of initial denaturation at 95°C, followed by 30 cycles of denaturation (30 s at 95°C), annealing (30 s at 57°C), and extension (1 min at 72°C), and final extension at 72°C for 7 min. The second-round amplification was conducted with 1 µl of the first-round PCR products as template and primers (nifH1 and nifH2)[1] under the same conditions.

Construction of non-redundant gene sets

The metagenomic reads were quality-filtered using a pipeline[2] and assembled into contigs using Newbler v2.7. The contigs ≥ 500 bp were subjected to gene prediction using MetaGeneAnnotator[3]. Non-redundant gene sets were constructed for the PNG and Japanese samples, respectively, with CD-HIT[4] using a 95% sequence similarity and a 90% length coverage as thresholds. The Japanese non-redundant gene set was combined with the set prepared from a published Japanese fecal metagenome[5].

Search for *nifH* in metagenomic sequencing reads

The metagenome sequencing reads from the samples used for the $^{15}N_2$ incorporation assay were further searched for *nifH*, *nifD,* and *nifK*, using MG-RAST (v3.2)[6] with the integrated microbial genome (IMG) database (https://img.jgi.doe.gov/cgi-bin/er/main.cgi). The following criteria were used: amino acid sequence identity greater than or equal to 80 %, E-value less than or equal to 10^{-5}, and 15 or more amino acids in length. The candidate reads were further screened by BLASTx searches: reads sharing 80% or greater sequence identity and positive score 90% or greater with known NifH sequences were selected.

Supplementary References

1 Zehr J P & McReynolds L A. Use of degenerate oligonucleotides for amplification of the *nifH* gene from the marine cyanobacterium *Trichodesmium thiebautii*. *Appl Environ Microbiol* **55**, 2522-2526 (1989).

2 Gomez-Alvarez V, Teal T K & Schmidt T M. Systematic artifacts in metagenomes from complex microbial communities. *ISME J* **3**, 1314-1317 (2009).

3 Noguchi H, Taniguchi T & Itoh T. MetaGeneAnnotator: detecting species-specific patterns of ribosomal binding site for precise gene prediction in anonymous prokaryotic and phage genomes. *DNA Res* **15**, 387-396 (2008).

4 Li W & Godzik A. Cd-hit: a fast program for clustering and comparing large sets of protein or nucleotide sequences. *Bioinformatics* **22**, 1658-1659 (2006).

5 Nishijima S *et al.* The gut microbiome of healthy Japanese and its microbial and functional uniqueness. *DNA Res* (2016).

6 Meyer F *et al.* The metagenomics RAST server - a public resource for the automatic phylogenetic and functional analysis of metagenomes. *BMC Bioinformatics* **9**, 386 (2008).

Table S1. Calculation of ^{15}N absolute mass and weight in faecal samples.

ID	Sample N ratio (%)	$^{15}N_2$ exposure (−)			$^{15}N_2$ exposure (+)			Nitrogen fixation rates
		$\delta^{15}N$(‰)	^{15}N(atom%)	^{15}N mg/ sample g	$\delta^{15}N$(‰)	^{15}N(atom%)	^{15}N mg/ sample g	^{15}N nmoles/ sample g/ h
V001	5.158	4.8277	0.3680	0.1898	5.7415	0.3683	0.1900	0.128
V004	1.846	3.0860	0.3674	0.0678	4.3825	0.3679	0.0679	0.065
V009	6.330	5.3287	0.3682	0.2331	6.1710	0.3685	0.2333	0.145
V014	4.900	5.3273	0.3682	0.1804	6.4357	0.3686	0.1806	0.147
V022	7.620	5.6390	0.3683	0.2807	6.3203	0.3686	0.2808	0.124
V035	5.650	2.6545	0.3672	0.2075	3.7380	0.3676	0.2077	0.146
J001	4.068	6.2177	0.3685	0.1499	7.4790	0.3690	0.1501	0.139
J002	5.340	4.7787	0.3680	0.1965	5.5830	0.3683	0.1967	0.117

To evaluate ^{15}N mass, ^{15}N absolute ratios (atom %) were calculated using the equation: A (atom %) = $100 \times [(^{15}N \times 10^{-3} + 1)(^{15}N/^{14}N)_{atmosphere}/1 + (\delta^{15}N \times 10^{-3} + 1)(^{15}N/^{14}N)_{atmosphere}]$, where $(^{15}N/^{14}N)_{atmosphere} = 0.03676$ and $\delta^{15}N$ = average value in replicates. Then, ^{15}N mass (^{15}N mg/sample g) was obtained by multiplying 'Sample N ratio' by '^{15}N absolute ratio'. 'Nitrogen fixation rates' (nmoles/sample g/h) were estimated based on subtraction of '^{15}N mg/sample g' values between the conditions '$^{15}N_2$ exposure (+)' and '$^{15}N_2$ exposure (−)'.

Table S2. *nifH* DNA and cDNA sequences identified from PNG and Japanese faecal samples.

	nifH DNA sequences										*nifH* cDNA sequences									
	PNG						Japanese				PNG						Japanese			
	V004	V014	V035	L005	L006	L015	J001	J002	J004	J005	V004	V014	V035	L005	L006	L015	J001	J002	J004	J005
OTU-01			29	9	4															
OTU-02																		14	14	19
OTU-03				1					13				6				6		6	
OTU-04				3										29						
OTU-05	7												1							
OTU-06											22	23								
OTU-07																	22			
OTU-08						3										17				
OTU-09					5	15														
OTU-10															20					
OTU-11							17													
OTU-12	8				1	7														
OTU-13	6												8							
OTU-14		17								13										
OTU-15																		12		
OTU-16		8	1		1	1														
OTU-17										10										
OTU-18				4	3	2														
OTU-19													5		4					
OTU-20					8															
OTU-21		4			4															
OTU-22									1								3		4	
OTU-23												7								
OTU-24	2							5												
OTU-25								5								2				
OTU-26							6													
OTU-27								5												
OTU-28									5											
OTU-29																5				
OTU-30								1			3									
OTU-31								4												
OTU-32													4							
OTU-33										2										2
OTU-34		1		3																
OTU-35															3					
OTU-36									3											
OTU-37				2				1	1											
OTU-38																				
OTU-39											1									1
OTU-40																	2			
OTU-41	2																			
OTU-42								2												
OTU-43								2												
OTU-44				2																
Total count	25	30	30	24	26	28	23	25	23	25	26	30	24	29	27	24	33	26	24	22

Table S3. PNG and Japanese individuals for metagenomic sequencing.

Sampling area and year	ID	Sex[a]	Age	BMI[b]	N intake[c]
Levani (2013)	V001	M	39	22.9	103.4
Levani (2013)	V004	M	22	22.7	109.6
Levani (2013)	V005	M	15	20.8	117.7
Levani (2013)	V009	M	35	24.3	117.2
Levani (2013)	V010	M	32	25.6	55.4
Levani (2013)	V014	M	22	26.2	67.3
Levani (2013)	V018	F	23	19.2	41.8
Levani (2013)	V020	F	24	24.9	46.2
Levani (2013)	V022	F	35	27.2	87.3
Levani (2013)	V031	F	27	23.4	74.7
Levani (2013)	V035	M	40	22.9	148.0
Levani (2013)	V036	F	36	19.4	81.1
Maprik (2012)	V038	F	25	23.2	65.5
Maprik (2012)	M025	M	33	23.3	68.7
Maprik (2012)	M031	F	29	22.6	115.5
Maprik (2012)	M101	F	22	19.2	66.3
Tokyo (2013)	J001	M	44	25.1	209.1
Tokyo (2013)	J002	M	40	21.6	183.2

[a] M, male; F, female.

[b] BMI, body mas index, given by body weight (kg) per body height(m)^2.

[c] Values were given by total N (mg) per body weight (kg) per day.

Table S4. *nifH* homologues identified in human faecal metagenome gene sets.

**Gene set**	**Identified homologues**	**Closest reference sequence**		**Identity %**	**Alignment length (aa)**	**E-value**
		**Species**	**Accession number**			
PNG	PNG-1	*Lachnospira multipara*	WP_031556311	94.4	213	8.12E-140
	PNG-2	Lachnospiraceae bacterium 3-1	EOS26764	97.6	123	6.65E-78
	PNG-3	*Clostridium* sp. CAG:75*	CCZ52034	97.9	146	7.47E-98
	PNG-4	*Clostridium* sp. CAG:75	CCZ52034	97.9	141	2.45E-94
Japanese	JPN-1	*Klebsiella pneumoniae* 342	AFV52053	99.4	179	9.17E-123
	JPN-2	*Clostridium* sp. CAG:75	CCZ52034	100.0	282	0.00E+00
Denmark_Spain_China	DSC-1	Lachnospiraceae bacterium 3-1	EOS26764	97.2	282	0.00E+00
	DSC-2	*Clostridium* sp. CAG:75	CCZ52034	100.0	282	0.00E+00
	DSC-3	Lachnospiraceae bacterium MC2017	WP_044906710	89.0	173	8.88E-105
	DSC-4	Enterobacteriaceae spp.	WP_004203553	100.0	293	0.00E+00
	DSC-5	*Clostridium* sp. CAG:75	CCZ52034	99.5	201	5.69E-139
Sweden	Sweden-1	*Clostridium* sp. CAG:75	CCZ52034	99.6	282	0.00E+00
	Sweden-2	Lachnospiraceae bacterium 3-1	EOS26764	97.2	282	0.00E+00
	Sweden-3	Lachnospiraceae bacterium 3-1	EOS26764	99.3	282	0.00E+00
	Sweden-4	*Butyrivibrio* spp.	WP_026655912	96.1	282	0.00E+00
	Sweden-5	*Butyrivibrio* spp.	WP_026655912	83.4	271	3.30E-166
	Sweden-6	Lachnospiraceae bacterium MC2017	WP_044906710	87.2	203	1.64E-123

* The data of *Clostridium* sp. CAG:75 is derived from human gut metagenome (BioSample: SAMEA3138515).

Table S5. *nifH* homologues identified from PNG and Japanese faecal metagenome sequencing reads.

Sample ID	Query sequence	Closest reference sequence		Identity %	Alignment length (aa)	E-value
		**Species**	**Accession number**			
PNG_V004	6INH6:01185:00221_1_153_+	*Clostridium beijerinckii*	WP_026886176	87.8	49	4.00E-21
PNG_V014	XK7CQ:00442:01839_1_278_+	Lachnospiraceae bacterium 3-1	WP_016286008	96.7	90	5.68E-55
PNG_V014	XK7CQ:01216:00792_1_240_-	Lachnospiraceae bacterium 3-1	WP_016286008	100	73	5.33E-41
PNG_V014	XK7CQ:00625:02773_1_346_-	Lachnospiraceae bacterium 3-1	WP_016286008	91.9	111	7.99E-62
PNG_V014	XK7CQ:01216:00792_1_240_-	Lachnospiraceae bacterium 3-1	WP_016286008	100	73	5.33E-41
PNG_V014	XK7CQ:03187:01948_1_161_+	*Clostridium* sp. CAG:75*	WP_004203553	98.0	49	4.91E-24
PNG_V022	Z28L8:00332:02004_1_324_+	Lachnospiraceae bacterium 3-1	WP_016286008	97.2	107	1.00E-66
PNG_V035	EU8EW:00090:01025_1_224_-	Lachnospiraceae bacterium 3-1	WP_016286008	98.4	61	3.00E-32
PNG_V035	SEU8EW:00502:02449_1_365_+	Lachnospiraceae bacterium 3-1	WP_016286008	98.2	108	1.00E-67
PNG_V035	EU8EW:00795:02054_1_240_-	Lachnospiraceae bacterium 3-1	WP_016286008	96.2	78	8.00E-45
PNG_V035	EU8EW:01823:00842_1_316_+	Lachnospiraceae bacterium 3-1	WP_016286008	97.1	104	3.00E-64
PNG_V035	EU8EW:02502:00267_80_316_-	Lachnospiraceae bacterium 3-1	WP_016286008	98.7	77	2.00E-47
PNG_V035	EU8EW:02796:01992_1_200_+	Lachnospiraceae bacterium 3-1	WP_016286008	96.9	65	7.00E-34
PNG_V035	EU8EW:03345:01041_1_232_-	Lachnospiraceae bacterium 3-1	WP_016286008	97.3	75	4.00E-41
PNG_V035	EU8EW:03452:01767_1_275_+	Lachnospiraceae bacterium 3-1	WP_016286008	97.8	91	1.00E-54
PNG_V035	EU8EW:01384:03160_1_194_-	*Butyrivibrio proteoclasticus*	WP_026651776	100	62	9.00E-33
PNG_V035	EU8EW:00304:01641_1_221_-	*Lachnospira multipara*	WP_031556311	96.2	52	5.00E-26
PNG_V035	EU8EW:00850:02075_1_355_-	*Clostridium* sp. CAG:75	WP_022024781	96.1	77	2.00E-44
PNG_V035	EU8EW:02766:01481_1_321_+	*Clostridium* sp. CAG:75	WP_022024781	97.1	104	1.00E-65
PNG_V035	EU8EW:01843:03240_1_149_+	*Clostridium* sp. CAG:75	WP_022024781	100	37	2.00E-15
PNG_V035	EU8EW:03175:03042_1_214_+	*Clostridium* sp. CAG:75	WP_022024781	100	69	8.00E-40
JPN_J002	41HRQ:01239:00544_1_229_+	*Klebsiella* spp.	WP_004203553	80.0	60	2.00E-19
JPN_J002	41HRQ:01239:00544_1_229_+	*Klebsiella* spp.	WP_004203553	98.7	75	9.00E-45
JPN_J002	41HRQ:02314:03199_1_181_-	*Enterobacter* spp.	WP_039078970	97.1	35	3.00E-13

* The data of *Clostridium* sp. CAG:75 is derived from human gut metagenome (BioSample: SAMEA3138515).

Table S6. *nifD* and *nifK* homologues identified from human faecal metagenome gene sets.

Gene set	Query sequence	Closest reference sequence			Identity %	Alignment length (aa)	E-value
		Definition	Species	Accession number			
PNG	V036_contig00879_gene_6_2543_2968_minus_0_11_39.5395_s	NifD	Ruminococcaceae bacterium AE2021	WP_028513096	96.2	133	6.79E-88
	V036_contig00879_gene_7_2946_3293_minus_0_11_18.0651_s	NifD	Lachnospira multipara	WP_027436974	83.5	97	4.74E-50
	V036_contig00879_gene_8_3290_3841_minus_0_11_43.5437_s	NifD	Lachnospira multipara	WP_031556307	86.7	181	4.23E-111
	V036_contig00879_gene_9_3790_3972_minus_0_11_6.7026_s	NifD	Ruminococcaceae bacterium AE2021	WP_028513096	93.5	46	2.13E-21
	V035_contig00800_gene_10_6894_7547_plus_0_11_36.3085_s	NifK	Butyrivibrio sp. AE3003	WP_026654166	81.5	211	1.61E-123
	V035_contig00800_gene_11_7604_8182_plus_0_11_50.996_s	NifK	*Clostridium* sp. CAG:75*	CCZ52039	80.2	192	6.69E-106
Japan	FTagent01_contig08661_gene_2_845_2053_minus_0_11_80.4959_b	NifD	*Clostridium* sp. CAG:75	CCZ52038	100.0	402	0.00E+00
	FTagent12_contig72540_gene_2_332_475_plus_0_10_8.24811_b	NifD	*Lachnospiraceae* bacterium 3-1	WP_016286010	91.7	48	1.94E-21
	Miseqsingleton_scaffold_193445_gene_1_1_319_plus_2_00_60.7152_b	NifD	*Klebsiella variicola*	WP_049165071	100.0	105	1.56E-68
	PGMsingleton_contig300576_gene_2_213_706_plus_0_10_44.649_b	NifD	*Klebsiella variicola*	|WP_044613654	87.2	156	5.23E-91
	re-singleton_contig113700_gene_1_1_228_minus_0_10_15.4132_p	NifD	*Klebsiella pneumoniae*	WP_046882228	93.4	76	5.01E-41
	FTagent01_contig08661_gene_1_8_826_minus_0_11_43.0035_b	NifK	*Clostridium* sp. CAG:75	CCZ52039	97.5	238	8.54E-166
	FTagent01_contig15875_gene_1_238_363_plus_0_11_4.57206_b	NifK	*Clostridium* sp. CAG:75	CCZ52039	100.0	41	3.80E-19
	Miseqsingleton_scaffold_199724_gene_1_1_181_minus_0_10_35.3352_p	NifK	*Klebsiella pneumoniae*	WP_061153956	100.0	60	1.05E-32
	re-singleton_contig101838_gene_1_1_557_plus_2_01_88.5988_b	NifK	Enterobacteriaceae	WP_008804133	100.0	184	1.78E-131
	singleton_HQWINQD02GQMQI_gene_1_1_597_minus_0_10_83.428_b	NifK	*Clostridium butyricum* 60E.3	ENZ32193	100.0	199	3.06E-143
	singleton_HQWINQD02GQMQI_gene_1_1_597_minus_0_10_83.428_b	NifK	*Klebsiella pneumoniae*	CAA29588	100.0	199	3.06E-143
Denmark_Spain_China	35118	NifD	*Clostridium* sp. CAG:75	CCZ52038	99.8	481	0.00E+00
	1818158	NifD	*Lachnospiraceae* bacterium 3-1	WP_016286010	88.7	467	0.00E+00
	35118	NifD	*Clostridium* sp. CAG:75	CCZ52038	88.8	480	0.00E+00
	3277371	NifD	*Klebsiella variicola*	WP_049165071	99.2	131	2.20E-89
	3280711	NifD	*Klebsiella variicola*	WP_044613654	99.7	335	0.00E+00
	3708105	NifD	*Clostridium* sp. CAG:75	CCZ52038	100.0	273	0.00E+00
	764122	NifK	*Butyrivibrio* sp. AE3003	WP_026654166	83.6	134	3.82E-75
	1797041	NifK	*Clostridium* sp. CAG:75	CCZ52039	90.0	50	1.04E-22
	1818155	NifK	*Butyrivibrio* sp. LB2008	WP_019553696	80.9	424	0.00E+00
	3273820	NifK	*Klebsiella pneumoniae*	WP_032740067	99.6	451	0.00E+00
	3323959	NifK	*Clostridium* sp. CAG:75	CCZ52039	98.7	394	0.00E+00
	3691959	NifK	*Lachnospiraceae* bacterium MA202	|WP_044922962	82.0	61	3.26E-27
	3708106	NifK	*Clostridium* sp. CAG:75	CCZ52039	99.0	96	9.74E-58
Sweden	gene_15804495_484_NODE_77899_length_17005_cov_6.235048	NifD	*Lachnospiraceae* bacterium 3-1	WP_016286010	88.7	486	0.00E+00
	gene_14996018_543_NODE_29503_length_19686_cov_7.427614	NifD	*Clostridium* sp. CAG:75	CCZ52038	99.8	481	0.00E+00
	gene_677624_317_NODE_122348_length_1171_cov_4.495303	NifD	*Lachnospiraceae* bacterium 3-1	WP_016286010	95.5	403	0.00E+00
	gene_18672520_88_NODE_16775_length_1066_cov_4.862101	NifD	*Clostridium* sp. CAG:75	CCZ52038	89.3	367	0.00E+00
	gene_15595724_243_NODE_77119_length_1084_cov_3.226937	NifD	*Clostridium* sp. CAG:75	CCZ52038	87.9	340	0.00E+00
	gene_10179291_195_NODE_43917_length_1157_cov_4.478825	NifD	*Clostridium* sp. CAG:75	CCZ52038	89.4	292	0.00E+00
	gene_9808769_552_NODE_116611_length_662_cov_2.649547	NifD	Butyrivibrio sp. MC2021	WP_026517309	89.8	49	2.14E-22
	gene_416326_NODE_2750038_length_11457_cov_23.302784	NifD	*Lachnospiraceae* bacterium 3-1	WP_016286010	95.5	490	0.00E+00
	gene_328325_NODE_2098381_length_3771_cov_9.653142	NifD	*Clostridium* sp. CAG:75	CCZ52038	90.2	256	4.73E-169
	gene_588445_NODE_4031947_length_6943_cov_22.383984	NifD	*Clostridium* sp. CAG:75	CCZ52038	88.5	480	0.00E+00
	gene_1581485_NODE_9865591_length_8092_cov_22.446737	NifD	*Clostridium* sp. CAG:75	CCZ52038	89.2	480	0.00E+00
	gene_10206795_195_NODE_77711_length_2199_cov_4.713506	NifK	*Butyrivibrio* sp. AE2005	WP_026668199	82.5	93	0.00E+00
	gene_18706167_88_NODE_48280_length_577_cov_3.233969	NifK	*Clostridium* sp. CAG:75	CCZ52039	82.4	125	9.82E-62
	gene_15801722_484_NODE_75170_length_6822_cov_6.351363	NifK	*Treponema* sp. C6A8	WP_027728473	89.3	112	9.70E-67
	gene_14139807_432_NODE_147117_length_1403_cov_3.354954	NifK	*Clostridium* sp. CAG:75	CCZ52039	99.0	101	3.21E-65
	gene_4311480_305_NODE_127346_length_1476_cov_4.300136	NifK	*Clostridium* sp. CAG:75	CCZ52039	100.0	60	1.78E-31
	gene_8870726_547_NODE_67519_length_13382_cov_21.034674	NifK	*Clostridium* sp. CAG:75	CCZ52039	90.0	50	1.46E-22
	gene_18022002_225_NODE_3657_length_13385_cov_22.584087	NifK	*Lachnospiraceae* bacterium MA2020	WP_044922962	82.0	61	3.07E-27

* The data of *Clostridium* sp. CAG:75 is derived from human gut metagenome (BioSample: SAMEA3138515).

Table S7. *nifD and nifK* homologues identified using MG-RAST from PNG and Japaense faecal metagenome sequencing reads.

**Sample ID**	**Query sequence**	**Closet reference sequence**			**Identity %**	**Alignment length (aa)**	**E-value**
		**Definition**	**Species**	**Accession number**			
PNG_V004	6INH6:02571:02398_1_353_-	NifD	*Raoultella terrigena*	AFV36358	99.0	115	3.00E-75
PNG_V004	6INH6:02764:01137_1_150_+	NifK	*Klebsiella* spp.	WP_016161448	98	47	3.00E-23
PNG_V014	XK7CQ:00908:00644_1_289_+	NifK	*Clostridium* sp. CAG:75*	CCZ52039	87.0	85	4.00E-52
PNG_V035	EU8EW:01327:00961_1_243_+	NifK	*Clostridium* sp. CAG:75	CCZ52039	81.0	74	5.00E-30
PNG_V035	EU8EW:01620:00672_1_337_-	NifK	Lachnospirceae bacterium MA2020	WP_044922962	94.0	104	7.00E-41
PNG_V035	EU8EW:02451:00193_1_280_-	NifK	*Clostridium* sp. CAG:75	CCZ52039	91.0	88.0	2.00E-42
PNG_V035	EU8EW:02954:00789_1_350_-	NifK	*Clostridium* sp. CAG:75	CCZ52039	88.0	92.0	7.00E-44
JPN_J002	41HRQ:01232:02393_1_337_-	NifD	*Klebsiella pneumoniae*	WP_060590496	100.0	47	5.00E-23
JPN_J002	41HRQ:02346:01381_1_343_-	NifD	*Klebsiella variicola*	WP_044613654	96.0	107	3.00E-67
JPN_J002	41HRQ:03302:02188_1_285_+	NifD	*Klebsiella variicola*	WP_044613654	97.1	93	2.00E-59
JPN_J002	41HRQ:00900:00564_1_210_+	NifK	*Klebsiella* spp.	WP_004203556	99	68	5.00E-39
JPN_J002	41HRQ:01033:02206_105_354_-	NifK	*Klebsiella* spp.	WP_016161448	95	79	6.00E-45

* The data of *Clostridium* sp. CAG:75 is derived from human gut metagenome(BioSample: SAMEA3138515).

Table S8. Quantitative PCR primers and probes (5′–3′)

Primers and probes	Sequence (5′–3′)
OTU-08 forward	ACCGTATTCTGAAAGAGGGATATGG
OTU-08 reverse	GGATGTGATAATTCCTCGGCCAG
OTU-08 probe	CATCCTACGCCTGGTTCTGGTCCTCCTG
OTU-24 forward	GGACTCCACCCGTCTGATC
OTU-24 reverse	CAACGGACATCGCCATAGC
OTU-24 probe	ACGTCTTCGAGCTCCAGATCCTCGAC
OTU-34 forward	TCGTCAGAGAAGGCTTTGGC
OTU-34 reverse	GCGTCCTGCACATCCAACA
OTU-34 probe	CGGTCCGCCGGATTCCACACATCTG

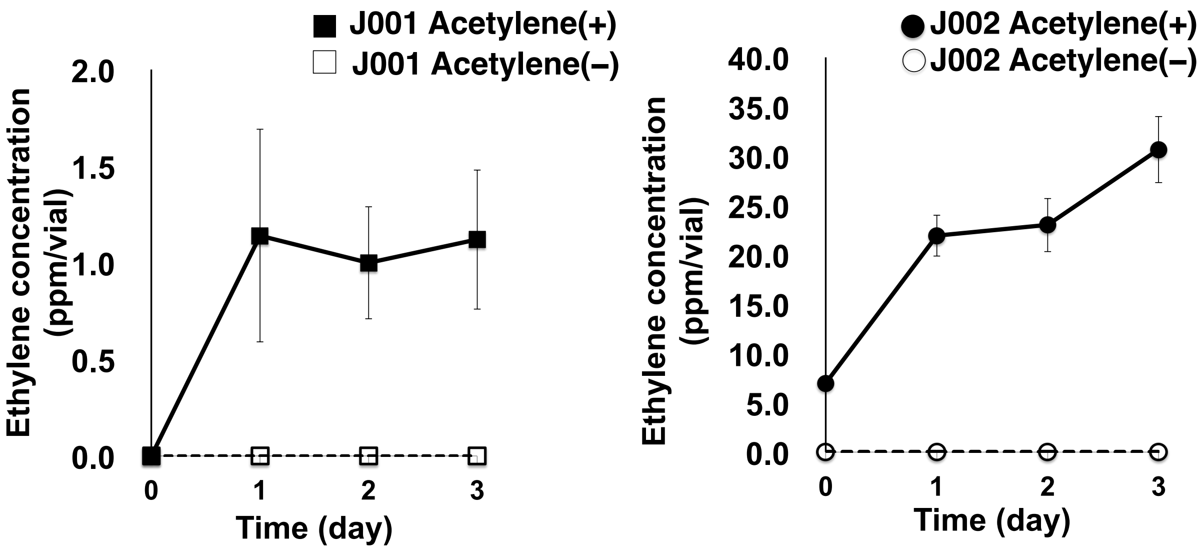

Figure S1.

Acetylene reduction assays in Japanese frozen fecal samples (J001 and J002).
